# Cellular Senescence in Immunity against Infections

**DOI:** 10.3390/ijms231911845

**Published:** 2022-10-06

**Authors:** Veronica Marrella, Amanda Facoetti, Barbara Cassani

**Affiliations:** 1 UOS Milan Unit, Istituto di Ricerca Genetica e Biomedica (IRGB), CNR, 20138 Milan, Italy; 2 IRCCS Humanitas Research Hospital, 20089 Milan, Italy; 3 Department of Biomedical Sciences, Humanitas University, 20090 Milan, Italy; 4 Department of Medical Biotechnologies and Translational Medicine, Università Degli Studi di Milano, 20089 Milan, Italy

**Keywords:** aging, senescence, exhaustion, immunity, infections, COVID-19

## Abstract

Cellular senescence is characterized by irreversible cell cycle arrest in response to different triggers and an inflammatory secretome. Although originally described in fibroblasts and cell types of solid organs, cellular senescence affects most tissues with advancing age, including the lymphoid tissue, causing chronic inflammation and dysregulation of both innate and adaptive immune functions. Besides its normal occurrence, persistent microbial challenge or pathogenic microorganisms might also accelerate the activation of cellular aging, inducing the premature senescence of immune cells. Therapeutic strategies counteracting the detrimental effects of cellular senescence are being developed. Their application to target immune cells might have the potential to improve immune dysfunctions during aging and reduce the age-dependent susceptibility to infections. In this review, we discuss how immune senescence influences the host’s ability to resolve more common infections in the elderly and detail the different markers proposed to identify such senescent cells; the mechanisms by which infectious agents increase the extent of immune senescence are also reviewed. Finally, available senescence therapeutics are discussed in the context of their effects on immunity and against infections.

## 1. Introduction

Cellular senescence is an irreversible state of cell cycle arrest and is triggered, in normal cells, by exposure to different stresses (oxidative stress, chemotherapeutic drugs, inflammation, oncogene activation, etc.) or irreparable damage. Originally described in fibroblasts, cellular senescence affects most tissues during aging, including lymphoid tissues, causing chronic inflammation and the deregulation of innate and adaptive immune functions. Senescent cells (SnCs) display key features, such as an enlarged nucleus, flattened morphology, a granular phenotype, increased production of lipofuscin, the activity of B-galactosidase (B-gal), the activation of cyclin kinase inhibitor proteins (e.g., p15INK4b, p16INK4a and p21Cip1) and resistance to apoptosis [1].

In addition, SnCs secrete chemokines, cytokines, metalloproteases, reactive oxygen species (ROS), metabolites, miRNAs and extracellular vesicles in a complex defined as the senescent secretory phenotype (SASP) [2,3]. With advanced age, SnCs accumulate in most tissues, likely due to their inefficient removal by the immune system [4,5]. An increased number of SnCs and the chronic secretome are the drivers of “inflammaging”, defined as persistent low-grade inflammation contributing to the enhancement of the frequency and severity of elderly pathologies [6,7]. Of note, the excessive generation of the SASP can also contribute to hyperinflammation (cytokine storm) associated pathogenic responses to stimuli [8].

Because of its numerous consequences on tissue homeostasis, cellular senescence is currently the focus of considerable interest as a pathway that could be targeted to ameliorate aging across multiple tissues [9]. In this review, we discuss the current understanding of age-related changes affecting immune cells and how they influence protective immunity to the most common pathogen infections in the elderly. Moreover, we summarize the strategies that are currently being explored for targeting senescent immune cells to improve immunity against infections.

## 2. Aging and Immunosenescence

The natural aging process differentially impacts all organ systems and results in the loss of organismal homeostasis, leading to increased mortality and morbidity in the elderly population. It is therefore the greatest risk factor for most chronic diseases [10]. The gradual decline in the immune system during aging is regarded as immunosenescence [11] and arises from two complementary processes: the direct effect of cellular senescence in immune cells and the indirect consequence of tissue cellular senescence. The latter is characterized by weakened organism barriers and fosters the release of various signaling molecules to which immune cells respond [12,13]. In this regard, Chambers et al. described how the recruitment of inflammatory monocytes induced by senescent fibroblasts inhibited tissue-resident memory T-cell activation and proliferation, impairing antigen-specific immunity [14]. This phenomenon of age-related immune remodeling leads to immune response dysregulation and deterioration, causing high susceptibility to infections, reduced vaccination response, deregulated inflammation and cancer [15,16].

Notably, the aged immune system can also induce senescence in peripheral tissues and cause tissue damage. A recent paper defines the contribution of immune system aging to organismal aging. The selective deletion of *Ercc1*, encoding a crucial DNA repair protein in mouse hematopoietic cells, resulted in the accumulation of spontaneous DNA damage and the premature onset of immunosenescence in mice, which was associated with increased levels of cellular senescence and damage in non-lymphoid organs [17]. Another study described T cells with dysfunctional mitochondria working as accelerators of senescence after selectively deleting the *Tfam* gene in CD4 and CD8 T cells [18]. T cells from mutated mice showed mitochondrial dysfunction, like those present in aged mice, and higher secretion of proinflammatory cytokines, resulting in premature inflammaging. Of note, these high levels of cytokines act as systemic inducers of senescence, contributing to several age-related impairments, including reduced responses to infections and cardiovascular and cognitive alterations. This suggests that senescent, aged immune cells have a causal role in driving systemic aging and therefore may represent a key therapeutic target to promote healthy aging.

**Figure 1:**
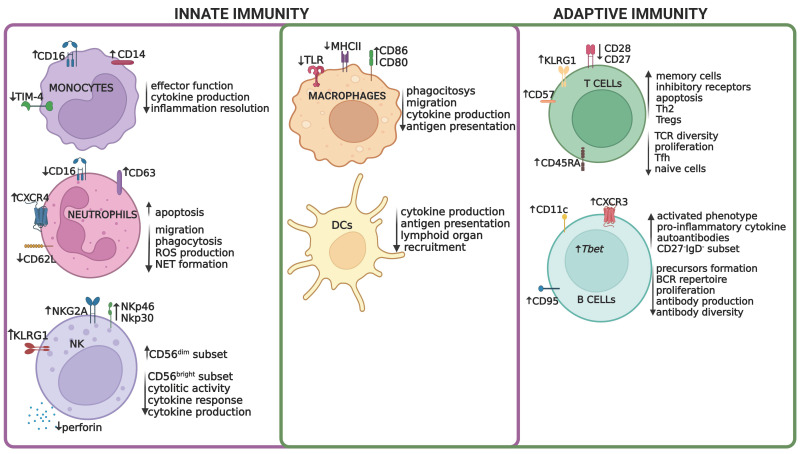
Simplified picture of phenotypical and functional modifications occurring in innate and adaptive immune cell populations during aging.

Although immunosenescence is mostly related to failures in adaptive immunity, aging also impacts various functions of the innate immune system, which in turn have been associated with failures in long-term humoral and cellular immunity. The characteristics of immunosenescence have normally been assessed through changes in surface marker expression and in the functional properties of different immune cell populations (Figure 1). Here, we summarize these phenotypic and functional alterations and report how they impact the overall response to infections.

### 2.1. Immunosenescence in the Adaptive Immune System

During aging, progressive thymic involution, linked to the reduced export of naïve T cells, is paralleled by an increase in antigen-experienced and memory effector T cells so that the absolute number of T cells is maintained [19,20].

However, while CD4^+^ T-cell blood counts are not affected, the CD8^+^ subset shows a strong decrease in the elderly [21]. Interestingly, the CD4/CD8 ratio value has been proposed as an integrative marker of biological age in older people, correlating with thymic output, phenotypic and functional alterations and general health status [21].

In humans, most T cells express the CD28 antigen. The proportion of CD28+ cells consistently decreases with age and is therefore considered a prognostic indicator of immunosenescence in the older population [22,23]. In addition, CD4^+^ and CD8^+^ T cells have been shown to express several inhibitory receptor molecules, including programmed cell death protein 1 (PD-1), lymphocyte activation gene 3 (LAG-3), cytotoxic T-lymphocyte antigen-4 (CTLA-4) and T-cell immunoglobulin and mucin-domain containing-3 (TIM-3), also associated with T-cell exhaustion [24]. Highly differentiated CD27-CD28-CD8+ populations display senescence features and can be further identified by the expression of Killer Cell Lectin-Like Receptor G1 (KLRG1) and CD57 markers and intracellular (MAP kinase p38 and γH2AX) molecules [25,26,27]. They also upregulate receptors associated with NK cells, such as inhibitory (NKG2A) and activator proteins (NKG2C and NKG2D) [28]. Recently, it was shown that Sestrins, stress-sensing proteins known to inhibit T-cell proliferation [29], are also responsible for the acquisition of this innate-like killing phenotype [30]. Human senescent T cells can also be characterized by the re-expression of the CD45RA antigen in highly differentiated T cells [31]. Moreover, senescent CD4 T cells expressing PD-1 and CD153 have been shown to accumulate in the spleens of aged mice [32].

T-cell receptor (TCR) diversity contracts with the increased risk of the generation of oligoclonal cell populations. In murine lymphocytes, studies have shown that TCR functionality diminishes with age, with reduced Signal Transducer and Activator of Transcription (STAT) phosphorylation, *cfos* expression and extracellular signal-regulated kinase (ERK) activation [33]. Impaired responses to stimuli in elderly T cells are associated with their pre-activation state, mirrored by the increased expression of CD25 and HLADR markers [34]. This also resulted in lower proliferative ability (reduced IL7 production) [35] and increased apoptosis (pro-apoptotic shift) [36].

In addition to shortened telomeres, DNA damage responses, constitutive MAP kinase activity and reduced proliferative activity, senescent T cells also exhibit a SASP profile, consisting of proteases and inflammatory cytokines (IL1β and TNFα) [37]. In humans, high SA-β-gal activity and p16 expression in T cells are considered reliable markers of senescence and are correlated with age and disease [38,39]. Similarly, in aged mice, Quinn et al. [40] reported a population of virtual memory CD8 cells displaying high p21 and gH2AX expression and reduced TCR-driven proliferation.

In the context of cytokine production, T-helper type 1 (Th1) cells, which secrete IL-2, IFN-γ and lymphotoxin, have a major role in cellular immunity to intracellular pathogens, virus infections and cancers, whereas Th2 cells, which secrete IL-4, IL-5 and IL-10, play a predominant role in humoral immunity against extracellular parasites [41]. A bias toward a Th2-type cytokine response (IL-4 and IL-10) has been shown to reflect the age-related decline in Th1 activation and differentiation [42]. Reduced differentiation toward T follicular helper (Tfh) cells has been reported as well and correlated with the reduced age-dependent expression of the transcription factor TCF1 [35,43,44]. Moreover, Tfh cells displayed reduced expression of the costimulatory molecule CD40L, which is important for a proper germinal center response [45]. Th17 cells were significantly increased in older individuals, whereas regulatory T cells (Tregs) were reduced. However, the overall Th17/Treg ratio decreased with age, which was associated with elevated Foxp3 mRNA and IL-10 protein expression [46]. These alterations might contribute to imbalanced proinflammatory and anti-inflammatory immune responses, which sustain a higher susceptibility to age-related inflammatory diseases [46]. Specifically in the elderly, augmented Treg function may favor tumor progression, chronic infections or tissue degeneration, whereas impaired Treg function may cause autoimmunity and/or chronic inflammation [47].

Several studies in mice indicated that pre-B, pro-B and immature B cells develop in lower numbers during aging [48], reflecting reduced levels of IL-7 in the bone marrow [48]. Interestingly, the impaired generation of pro-B cells has also been linked to TNF-α, secreted in high amounts by proinflammatory “age-associated B cells” (ABCs), which populate the bone marrow of old mice [49]. Two groups described ABCs in the spleens of aged mice [50,51]: these cells displace FO B cells with advancing age, are refractory to BCR but TLR7/TLR9-sensitive and have a high propensity to proinflammatory and regulatory cytokine production (IFN-γ and TNF-α) [50,51]. Accordingly, they are able to skew T-cell polarization toward an inflammatory subset, suggesting a role for ABCs as players in basal inflammatory states associated with aging [52]. In humans, B-cell percentages and numbers significantly decrease with age [53]. Regulatory B cells (Bregs) are potent suppressors of immune responses, both through the secretion of IL-10 and through other contact-dependent mechanisms [54]. It has been shown that these cells decrease with age and maintain unaltered TNF-α production, while the production of IL-10 declines significantly, correlating with the appearance of autoantibodies [55,56]. Aged B cells produce fewer antibodies with lower affinity since affinity maturation by hypermutation is disturbed as well. This results in the reduced quality and quantity of antibody production in response to infection [57]. There is a shift in the proportions of the different B cell subsets: aging induces a significant decrease in the percentage of switched memory B cells and a significant increase in the percentage of naive and CD27^−^IgD^−^ double-negative (DN) late memory (LM) B cells, whereas no changes were observed in IgM memory [58,59]. In other reports, IgM memory B cells were found to be reduced in the elderly, resulting in a predisposition to pneumococcal infection [60,61]. In older people, DN LM B cells share similar characteristics with the above-mentioned murine ABCs. They are the most proinflammatory B-cell subset, which has also been described in the blood of patients with infectious diseases [62,63,64]. In the elderly, the CXCR3 and CD11c markers are expressed at high levels, indicating their ability to migrate to inflamed tissues and to contribute to local inflammation [58]. DN LM B cells affect the microenvironment by secreting proinflammatory mediators that, in turn, amplify the inflammatory response [59]. Moreover, they display senescence features, such as a poor ability to proliferate, reduced telomerase activity, high levels of SASP factors and elevated p16 expression [65,66].

### 2.2. Immunosenescence in the Innate Immune System

Aging results in altered numbers and functions of different innate immune cell subsets. Neutrophils are the first cells to reach the sites of infection. The number of circulating neutrophils does not change in the elderly compared to young people, but their function is significantly compromised [67]. Neutrophil adhesion to the endothelium appears to be unaltered in the aging population [68,69], suggesting that the extravasation of neutrophils is not affected. Activated neutrophils, in both aged humans and mice, are more prone to apoptosis and less effective in phagocytosis, chemotactic activity, and ROS production [70,71,72]. Interestingly, impaired neutrophil migration has been linked to increased constitutive PI3K signaling, and PI3K-blocking strategies were able to restore neutrophil chemotaxis. Targeting PI3K signaling may therefore be beneficial in improving neutrophil functions during infections and reducing inappropriate inflammation in older patients [73]. Aged neutrophils have been characterized by the increased expression of CXCR4 and the low expression of CD62L markers [74]. Increased degranulation is supported by the higher surface expression of CD63 and increased neutrophil elastase degradation products [73]. There is a reduction in the phagocytic ability for opsonized bacteria [69,71], especially for *Staphylococcus Aureus* [75], to which the elderly are particularly susceptible. The CD16 marker is reduced with age, impacting Fc-receptor-mediated phagocytosis and the oxidative burst [71]. In contrast, the phagocytic deficit for unopsonized bacteria does not seem to be compromised [76]. Moreover, aged mice have been demonstrated to have impaired neutrophil extracellular trap (NET) formation, leading to the increased dissemination of *S. Aureus* [77]. Finally, NET release in response to several physiological and pathological stimuli, such as lipopolysaccharide (LPS) and interleukin-8 (IL-8), was reduced in cells isolated from old subjects [78].

Natural killer (NK) cells have the key ability to produce cytokines after cell recognition, thus having an important part in innate immunity during aging. Several studies have reported changes in the absolute number of circulating NK cells as well as their subset distribution during aging [79]. In contrast, NK progenitor numbers in the peripheral blood or in the bone marrow are not affected [80]. Nonetheless, most studies reported an increase in the mature NK cell number, associated with a decline in the CD56bright subset and a concomitant increase in the CD56dim subset, reflecting reduced cytolytic activity and a reduced ability to produce cytokines [81,82,83,84]. The expression of NK cell receptors NKp46, NKp30, KLRG1 and NKG2A has been shown to decline with age, whereas NKG2D and CD16 expression remains unaltered [83]. NK cell cytotoxicity (NKCC) mediated by perforin exocytosis is reduced with age [81,82]. Indeed, defects in the polarization of perforin have been described at the immunological synapse between NK and cancer cells [81]. In contrast, NK-cell-mediated antibody-dependent cell cytotoxicity (ADCC) is preserved with age [85]. Persistent NK cell signaling via CD158d-HLA-G interaction induced a DNA damage response pathway, leading to senescent NK cells expressing p21 and secreting proinflammatory and proangiogenic mediators [86].

Macrophages create a heterogeneous population with different phenotypes and behaviors, depending on their anatomical location [87]. Studies in aged mice showed augmented myeloid progenitor cells in the bone marrow [88] and an increased number of macrophages in the spleen and bone marrow [89]. Conversely, mono/macrophage cells are reduced in the blood and in the bone marrow of older adults [90]. Aged macrophages from both humans and mice showed the reduced expression of MHCII molecules, which could limit T-cell responses in the elderly [91].

During aging, macrophages may present reduced phagocytic activity [92], reduced TLR expression [93] and altered cytokine responses to LPS [94]. During their activation, macrophages can polarize into M1 macrophages, which are capable of proinflammatory responses producing cytokines such as IL-6, IL-12 and TNF-α, or into M2 macrophages, which exert anti-inflammatory responses and are involved in damaged tissue repair [95]. There are reports suggesting that macrophage polarization is affected during aging: the nature of skewing is still controversial, but perturbations in the normal M1-M2 balance might dysregulate the host immune response, contributing to increased susceptibility to inflammatory and infectious diseases [96,97,98]. In line with this, the observed reduction in TLR expression in the elderly might be responsible, at least in part, for the higher susceptibility to bacterial, mycotic and viral infections [89]. On the contrary, Qian et al. reported a significant increase in the TLR5-induced production of IL-8 in monocytes from older individuals, accompanied by the increased expression of TLR5 and the phosphorylation of downstream MAPK, suggesting that targeting TLR5 may be a notable mechanism to enhance immune responsiveness in elderly [99]. In vitro studies have shown that human macrophages subjected to external stressors develop p21- and p53-mediated cell cycle arrest, elongated morphology and increased B-gal activity [100]. Some studies have reported a characteristic SASP in macrophages [101,102]. Interestingly, macrophages, which are naturally fusogenic, are primarily involved in senescent giant cell formation, which is a hallmark of chronic inflammation. Senescent multinucleated giant cells with their secretomes are a source of inflammation in aging arteries and gonads. These cells can revert their senescence state, leading to cancer progression and metastasis [103].

In the context of antigen-presenting cells, monocyte subsets did not reveal significant age-related alterations under physiological conditions; however, upon stimulation, elderly monocytes showed reduced production of IFN-α, IFN-γ, IL1-β, CCL20 and CCL8 and increased CX3CR1 [104,105]. In particular, nonclassical proinflammatory CD14+CD16+ monocytes [106] accumulate with age and show senescence features such as telomere shortening, reduced proliferation and SASP secretion [107]. In addition, a recent study revealed that aging alters critical pathways important in the production of type I interferon in CD14^dim^CD16^+^ monocytes, resulting in impaired innate immune responses to pathogen recognition receptor (PRR) agonists [108]. The frequency of circulating myeloid-derived suppressor cells (MDSCs) increases with age in both humans and mice [109]. Interestingly, proinflammatory factors produced by senescent cells promote both the proliferation and activation of MDSCs, which, in turn, secrete the anti-inflammatory cytokine TGF-β, a potent inducer of cellular senescence in inflamed tissues [110].

Dendritic cells (DCs), as macrophages, represent a bridge between innate and adaptive immunity. The numbers of circulating plasmacytoid (pDCs) and conventional (cDCs) dendritic cells are reduced in healthy and frail elderly people [111], while their distribution in different tissues seems to be unchanged [112]. Specifically, the number of skin Langherans DCs has been shown to decrease with age [113]. Several reports have documented that pDCs from elderly donors produced lower levels of IFN-γ in response to TLR ligands [111,114]. Despite the limited data available, DCs seem to be compromised with aging, helping to explain several phenotypes of immunosenescence (increased susceptibility to infection, loss of tolerance and lower vaccination response) [115].

## 3. Immune Response to Infection in Aging

Older adults exhibit increased susceptibility to infections and their complications. For this reason, vaccinations are strongly recommended in the elderly, but because of age-related changes in immune functions, vaccine efficacy and effectiveness also decline with age [116]. Influenza virus is one of the most studied viruses in relation to changes that occur with aging. Murine models of aging and influenza infection revealed dysregulated innate immune responses to viruses. Neutrophils accumulated in the lungs of aged mice as early as day 1 post-infection, and this increase was maintained throughout the course of infection as compared with young mice and correlated with higher pulmonary CXCL1 and CXCL2 levels [117]. Likewise, during influenza infection, aged rhesus macaques exhibited increased pulmonary levels of IL-8, inducing neutrophil chemotaxis, compared with younger macaques [118]. Interestingly, whereas neutrophil depletion at the time of infection increased mortality, depletion occurring during later stages improved survival in aged mice and reduced the levels of proinflammatory cytokines [117], suggesting that excessive neutrophil retention in the lung during infection is detrimental. Consistently, high levels of neutrophil-related transcripts in whole blood were correlated with severe outcomes and morbidity in individuals with influenza infection [119,120]. Alveolar macrophages (AMs) play a crucial role in viral clearance and in inflammation resolution during influenza infection, and aging severely impacts these functions. The adoptive transfer of AMs from young mice into the airways of aged mice has been shown to reduce lung tissue injury upon influenza infection [121]. A diminished ability to clear apoptotic cells from the lung environment could further contribute to inflammation and immune system dysfunction. Similar findings have been reported in humans [122,123]. Adaptive immune responses to influenza infection are also impaired during aging. Reduced numbers of both CD4^+^ and CD8^+^ influenza-specific pulmonary T cells were reported during influenza infection in aged rhesus macaques and mice [118,124]. Additionally, virus-infected aged mice generated a restricted repertoire of influenza-specific T cells, mainly in the CD8^+^ compartment [125,126]. Likewise, alterations in the TCR repertoire have also been documented in older people [127], with important implications for vaccination. In mice, aging delayed T-cell infiltration into the lungs following influenza infection [128,129], and adoptive transfer experiments indicated that this defect might be partially due to the aged microenvironment, though the exact mechanisms are not well understood [130,131]. On the other hand, the reduced cDC priming of T cells might play a role [132]. Following influenza infection, cDCs similarly displayed delayed infiltration kinetics into the lungs of aged mice [129] and impaired migration to the lung dLN [133]. The age-associated defect in DC migration to lymph nodes could be ascribed to both environmental and cell-intrinsic factors [132,134,135]. In the context of B-cell responses, the production of influenza-neutralizing Abs was found to be compromised in aged mice, rhesus macaques and humans [118,129,136]. Reductions in Abs may reflect intrinsic defects or reduced CD4^+^ T-cell help [131,136,137]. Diminished Ab titers during influenza infection could also explain the reduced efficacy of vaccines in the elderly [138].

Respiratory syncytial virus (RSV) leads to rates of infection and disease severity comparable to influenza in older people. In mice, aging impaired the RSV-specific CD8+ T-cell response [139]. The viral load was higher in aged mice even if they displayed higher pulmonary levels of antiviral type I IFN signaling [139,140]. Aged humans have reduced numbers of RSV-specific CD4^+^ and CD8^+^ T cells and reduced IFN-γ secretion by RSV-specific T cells [141]. In addition to influenza and RSV, other respiratory viruses, such as rhinovirus, metapneumovirus and parainfluenza, cause severe burdens in older people [142,143]. The impact of aging on the immune response to these respiratory viruses is understudied and thus not well understood. However, the findings in RSV or influenza models may inform on these other respiratory viruses, since they activate common immune pathways, such as type I IFN and Th1 responses.

The bacterium *Streptococcus pneumoniae* is also a common cause of severe pneumonia in the elderly, often leading to sepsis [144]. In response to pneumococcal pneumonia, aged mice displayed increased neutrophil recruitment in the lung, even though this did not affect protective immunity [145]. Likewise, a higher percentage of neutrophilic granulocytes was immunohistochemically quantified in lung tissue specimens from the elderly compared to young patients with microbiologically proven *S. pneumoniae* pneumonia [146]. Conversely, young patients versus elderly patients had more alveolar macrophages [146]. The activation of c-Jun N-terminal kinase/activator protein-1 (JNK/AP-1), one of the MAPK signaling pathways, was significantly upregulated in response to *S. pneumoniae* infection in PMNs from old mice compared to young controls, and its pharmacological inhibition reversed the defect in pneumococcal killing by PMNs, indicating that this pathway can potentially be targeted to reverse the age-related dysregulation of PMN responses [147]. Lower IL-10 production was associated with higher levels of chemokine ligands CXCL9, CXCL12, CCL3, CCL4, CCL5, CCL11 and CCL17 in infected old mice, suggesting a defect in anti-inflammatory cytokine production by immune cells in the lung [145]. The neutralization of IL-10 in infected young mice was associated with increased neutrophil recruitment but no decrease in bacterial outgrowth, similar to what was observed in aged mice [145]. A decrease in proinflammatory cytokine (TNF-α, IL-1β and IL-6) production by macrophages from aged mice has been observed after pulmonary infection with *S. pneumoniae*, suggesting that the reduction in TLR signaling, especially TLR2, occurs in vivo during an active infection [148,149]. Other functional impairments observed in aged macrophages during *S. pneumoniae* infection include the decreased expression and function of the NLRP3 inflammasome [150] and LC3-associated phagocytosis [151]. Alterations in marginal zone macrophages and marginal zone B cells were also reported in old mice [151,152], which may affect the ability to clear blood-borne antigens and mount proper T-independent immune responses. This is consistent with the evidence of reduced serum levels of anti-pneumococcal IgM in elderly persons compared with those in adults after pneumococcal vaccination [61]. Furthermore, declines in sTNFR-I and in the TNF-α/IL-10 ratio during pneumococcal infections were correlated with age in humans [153].

## 4. Immune Senescence Driven by Pathogen Exposure

In addition to natural aging, chronic infections induce a phenotype in immune cells that shares features with immunosenescence [154]. After the primary infection, many viruses can evade the immune system and persist in the host. In some circumstances, these infections become chronic because, due to the lack of immune containment, the virus continuously replicates, generating persistent antigenic stimulation for several months or even decades. Other chronic infections become latent, where very little viral replication occurs and almost no systemic viral antigens are present or hide from the immune system. Many latent infections, however, can become reactivated (i.e., caused by stress), leading to productive viral replication and the increased systemic presentation of viral particles [155]. The antigenic overload imposed by persistent infection and latent viruses exposes the immune system to permanent stress that leads to host T-cell exhaustion [156]. T-cell exhaustion was first described in mice during chronic lymphocytic choriomeningitis virus (LCMV) infection [156], but exhausted T cells have also been observed during infection with simian immunodeficiency virus (SIV) [157] and in individuals infected with a multitude of chronic human viruses. Unlike normal memory cells, the proliferation of exhausted T cells is only sustained by the presence of the viral antigen, partly due to losses of interleukin-2 receptor-b (CD122) and interleukin-7 receptor (CD127) [158,159]. Because the viral antigen is constantly or intermittently supplied, viral-specific T cells never cease proliferating. These dysfunctional memory T cells lack telomerase, and thus, the progressive erosion of chromosome telomeres, induced by repeated rounds of cell division, triggers mechanisms for replicative senescence, leading to premature cellular proliferative arrest [160]. Paradoxically, telomere erosion is coupled with increased resistance to apoptosis, allowing these senescent T cells to accumulate in the blood and tissues at the expense of functional naïve T cells, leading to biological aging and impaired immunity [161]. During chronic viral infections, CD8^+^/CD28^−^ T cells have reduced expression of effector molecules (granzyme B and perforin) and reduced cytotoxic T-lymphocyte (CTL) activity [162]. Regulatory T cells (Tregs) also have altered functions during chronic viral infections, particularly enhanced proliferation and immune-suppressive actions, as highlighted by the upregulation of PD-1 and CTLA-4 markers on their cell surfaces [163,164].

Critical telomere shortening has been shown to contribute to persistent DNA damage and the consequent induction of the DNA damage response (DDR); if DNA damage is not repaired, either programmed cell death (apoptosis) or the irreversible loss of division potential (replicative senescence) occurs. In the absence of apoptosis, cells harboring dysfunctional telomeres are at risk of developing genomic defects. Although, in some cases, apoptotic markers have been found in T cells progressing to senescence, replicative senescence is permanent. In vitro studies have shown that repeated antigenic stimulation led to activation-induced cell death resistance [165]. These cells increased PI3K/AKT signaling through Phosphatase and Tensin Homolog (PTEN) loss, leading to impaired CD95 activation. Long-term antigen exposure also increased the intracellular expression of Bcl-2-Like Protein-1 (Bcl-xl) and reduced the expression of the pro-apoptotic proteins Bad and Bax. The choice of apoptosis versus replicative senescence may be dependent on the state of cell differentiation, with less differentiated states (CD27+) more prone to apoptosis and late differentiated cells (CD27−) more prone to replicative senescence.

### 4.1. Immune Senescence during Chronic Persistent Viral Infections

Although antiretroviral therapy for human immunodeficiency virus (HIV) infection reduces AIDS morbidity and mortality, it does not fully restore health. Long-term-treated patients retain a higher risk of a number of complications, partially related to HIV-associated immunologic dysfunction [166]. Indeed, it is recognized that, with antiretroviral therapy (ART), HIV-specific T cells are exposed to viral antigens for a longer period, potentially causing increased T-cell exhaustion and immune senescence. Consistent with this evidence, aggressive forms of the disease present more cells with markers of T-cell exhaustion [167]. In HIV-infected individuals, the enhanced expression of inhibitory markers (PD-1, CTLA-4 and LAG-3), low CD127, reduced proliferative and self-renewal potential, impaired production of effector cytokines and CD4+ T-cell help have been widely documented [168]. Remarkably, CD8^+^ T cells also bear an exhausted phenotype in HIV patients and, in contrast to CD4^+^ T cells, which retain their replicative potential despite undetectable telomerase activity, become immunosenescent [169]. Telomerase addition resulted in increased viral targeting and reduced p16 and p21 levels [170].

Like HIV, Hepatitis B and C (HBV and HCV) viruses are persistent chronic viruses, leading to T-cell exhaustion from telomere shortening. Exhausted HCV-specific T cells have been found in the liver, spleen, and blood. In HCV-specific T cells expressing low CD127 levels and high levels of inhibitory markers (PD-1, TIM-3 and CD57), impaired proliferation and CTL responses were reported [171]. During chronic HCV infection, exhausted T cells undergo senescence and show all hallmarks of DNA damage, including γ-H2AX and p53 serine phosphorylation. Immune impairment was seen in CD8^+^ γ-H2AX^+^ T cells, which failed to signal through the JAK/STAT pathway [172].

The presence of exhausted T cells is also reported in patients suffering from severe acute respiratory syndrome coronavirus 2 (SARS-CoV-2) infection. Along with this, inhibitory receptors (TIM-3 and PD-1) are also detected in a subpopulation of T cells isolated from hosts infected with SARS-CoV-2 [173].

### 4.2. Immune Senescence during Chronic Latent Viral Infections

Hallmarks of T-cell exhaustion have been found in two latent viruses, VZV and herpes simplex-1/2 virus (HSV-1/2). VZV-specific T cells declined with age, lost T-cell functions and expressed T-cell exhaustion markers [174]. Exhausted CD8^+^ T cells have been documented in in vivo models [175]. However, telomere lengths in VZV- and HSV-specific T cells were not significantly shortened or senescent, probably due to the viruses’ ability to remain hidden from immune surveillance due to their location in neurons [176].

On the contrary, human T-cell leukemia virus type-1 (HTLV-I) predominantly infects immune cells and CD4^+^ T cells. However, there is evidence that replicative senescence occurs in HTLV-I-specific CD8^+^ T cells. HTLV-I is responsible for two different diseases: a fatal T-cell cancer developing after a very long latency period, Adult T-cell Leukemia/lymphoma (ATLL), and a neurodegenerative disease, Tropical Spastic Paraparesis/HTLV-I-associated myelopathy (TSP/HAM) [177]. T cells from both ATL and TSP patients display exhaustion markers, including PD-1, TIGIT, TIM-2 and 2B4 [178,179]. T cells from TSP patients were also deficient in CD28 and CD27 expression [180]. Senescent CD8^+^ T cells showed impaired proliferation and increased levels of proinflammatory and pro-senescence cytokines, such as IL-6 and TNFα. Total PBMCs from ATL patients have defects in telomerase expression and harbor shortened telomeres. These are recognized as DNA double-strand breaks and activate the DDR pathway, elevating ATM and p53 activities and culminating in replicative senescence [181].

Epstein–Barr virus (EBV) is one of the most common human viruses in the world and is responsible for a wide spectrum of human diseases, including those associated with defective B cells, such as acute infectious mononucleosis (AIM), and a variety of non-B-cell diseases. Moreover, HTLV-I is oncogenic. Studies have reported high PD-1 and TIM-3 expression on EBV CD8^+^ T cells from healthy individuals, which were increased in EBV/HIV-co-infected individuals [182]. EBV-specific exhausted T cells have also been found in EBV-associated diseases, such as chronic fatigue syndrome (CFS), multiple sclerosis (MS) and systemic lupus erythematosus (SLE). Signs of T-cell exhaustion encompassed elevated PD-1 and decreased cytotoxic T-cell functions in EBV CD8^+^ T cells from SLE patients, diminished T- and B-cell memory responses in CFS and reduced EBV CD8^+^ T-cell functionality and exhaustion in MS [183,184]. Uncontrolled chronic viral exposure leads to replicative senescence in EBV CD8^+^ T cells, as also reported in HIV-infected immunocompromised individuals and in X-linked lymphoproliferative syndrome (XLP) [185,186,187].

Human cytomegalovirus (CMV) is a member of Herpesviridae, infecting ~50% of the developed world’s population. The immune response elicited to control the primary infection involves natural killer (NK) cells, inflammatory cytokines, B cells and T cells, but CMV often persists, establishing a latent infection with periodic reactivation. During infection, CMV-specific CD8^+^ T cells undergo massive clonal proliferation and expand into as much as the totality of memory CD8^+^ T cells. These cells display an altered phenotype and are dysfunctional, likely representing a stage in the transition to replicative senescence [188]. Expansion also occurs in CMV-specific CD4^+^ T-cell populations, but to a much lesser extent [189]. The CMV-specific memory T cells are CD28^−^CD27^−^CD8^+^ and do not rely on costimulatory signals for their activation [190]. Increased CD57 expression and the loss of CD122 and CD127 have also been documented [191]. The occurrence of telomere shortening presumably limits the proliferative potential of CD28-CD57^+^ antigen-specific T cells. Antigen-exposed CMV-specific CD8^+^ T cells can simultaneously produce IFN-γ and TNF-α more quickly and at higher levels than other virus-specific CD8^+^ T cells [192], evocative of the secretory features (SASP) typical of replicative T-cell senescence.

T-cell exhaustion leading to immune senescence has been less characterized during some other chronic viral infections, such as human herpesvirus-8 (HHV-8) and human papilloma virus (HPV) infections. Senescence markers, such as increased CD57^+^/CD28− on CD4+ and CD8+ T cells and PD-1 on NK cells, have been found in HHV-8-infected individuals with HIV-associated or classic Kaposi’s sarcoma [193,194]. T-cell exhaustion may not occur in HPV to the same extent as in other infections due to its low antigenic loads. Otherwise, it is difficult to detect the possible localization of HPV-specific T cells to the mucosal tissue. In high-grade HPV infection, T cells and dendritic cells from cervical tissue showed positivity for the PD-1 marker. In addition, reductions in IFN-γ and IL-12 from helper T cells were observed [195]. Finally, other helper cells, including DCs, become dysfunctional in the context of chronic infections. DCs isolated from patients with HIV, HBV and HCV infections exhibited a diminished ability to produce cytokines and to induce T-cell activation. Such reductions impaired the CD8 T-cell antiviral response and drove exhaustion [196].

### 4.3. Immune Senescence during Bacterial Infections

Like viruses, many bacteria have the ability to circumvent immune surveillance and establish chronic infections, which, in some instances, may also evolve in host cell transformation and cancers. Evidence suggests that, to establish infections, pathogenic bacteria elicit DNA damage in host cells, but the effect on host immune cells remains poorly investigated. Mathiasen et al. recently reported that genotoxins, cytolethal distending toxins (CDTs) produced by a wide range of Gram-negative bacteria, including *Helicobacter hepaticus, Escherichia coli* and *Shigella dysenteriae*, induce DNA damage, DDR activation and premature senescence in activated CD4 T cells both in vitro and in vivo. The CDT-induced SASP phenotype in senescent CD4 T cells was partially orchestrated by ATM-p38 signaling [197]. The study, however, did not address whether these senescent T cells are long-lived; further studies are needed to assess whether they eventually die from the cytotoxic effect of the toxin or are cleared by immune-mediated destruction. *Salmonella* pathogens rely on typhoid toxin (TT) for dissemination and pathogen colonization. Another recent work revealed that TT induces a non-canonical DDR in macrophages that drives a senescence-like phenotype characterized by the accumulation of γH2AX and SASP [198]. Importantly, the SASP caused transmissible senescence in bystander cells, which became, in turn, more susceptible to *Salmonella* infection [198]. These findings demonstrate that pathogenic bacteria exploit senescence to dysregulate the host’s immune response and to promote a favorable niche for successful infection. Many questions regarding bacteria-induced senescence remain to be addressed, including the detailed analysis of the markers of immune senescence and of the SASP and whether they would be different from those induced in the context of viral infections. The current knowledge is limited and mostly related to infections with *Mycobacterium tuberculosis* (Mtb). Murine studies have demonstrated that chronic Mtb infection is associated with the differentiation of CD4 T cells toward an exhausted memory phenotype, with high expression levels of KLRG-1 and IFN-γ-monofunctional capacity and poor ability to contribute to antigen clearance [199]. Human Mtb-specific CD4^+^ and CD8^+^ T cells ex vivo display reduced production of INF-γ, TNF-α and IL-2. In addition, increased expression levels of PD-1 and its ligands are found on T cells, neutrophils, monocytes, macrophages and B cells from patients with active pulmonary tuberculosis [200]. The blockade of PD-1/PD-L1 signaling could enhance the specific degranulation of CTLs and restore specific IFN-γ activity [201,202]. This is in accordance with the fact that PD-1 and PD-L1 knockout (KO) mice display increased susceptibility to infection [203]. LAG-3 and TIM-3 are increasingly expressed on CD8^+^ T cells during the chronic phase of infection if not supported by the CD4^+^ compartment [203].

Studies in patients experiencing HIV-Mtb co-infection confirm that Mtb plays a major role in accelerating HIV disease progression by directly or indirectly facilitating factors associated with immune senescence. In fact, Mtb enhanced the expression of CD38, CD57, CD69, human leukocyte antigen-DR and the downregulated CD28, CD27, CD40L and CD127 markers on HIV-specific T cells [204]. Interestingly, T cells with a senescent phenotype (high expression of CD57, KLRG-1 and γH2AX and short telomeres) accumulate during cutaneous infections with the protozoa *Leishmaniasis braziliensis*, contributing to the inflammatory response in this disease [205]. This suggests that the cell fate of immune senescence might be a common endpoint in infections elicited by different pathogen species other than viruses. Future efforts providing mechanistic insights into the immunomodulatory properties of these pathogens and the link to immune senescence will be important in developing therapeutic strategies to eradicate them.

## 5. Immunometabolism and Cellular Senescence

Age-related alterations in metabolic homeostasis and nutrition have been linked to changes in different physiological functions, including immune functions. In this regard, the recent literature suggests that age-associated dysfunctional metabolism, driven by the accumulation of key host metabolites (saturated fatty acids, cholesterol, ceramides and lactate) and the loss of others (glutamine, tryptophan and short-chain fatty acids), might play a role in driving immune senescence and inflammaging and ultimately lead to diseases in the elderly [206]. Furthermore, the reduced availability of any one of these metabolites could negatively impact the magnitude of the antiviral response. Despite their proliferative arrest, immune cells with a senescent phenotype show high metabolic activity to cope with the high energetic demand of the senescence program, including SASP-coupled proteotoxic stress [207,208]. Generally, senescent immune cells choose glycolysis instead of OXPHOS, leading to an unbalanced bioenergetic condition caused by the accumulation of dysfunctional mitochondria. The consequent Ca++ buffering deficits found in aged mitochondria have the effect of reducing Ca++-mediated signaling in the target cells. Senescent T cells produce less ATP, with the consequences of the defective induction of several metabolites and reduced T cell activation. Specifically, the loss of the CD28 antigen has been associated with the loss of metabolic fitness. The preferential use of glycolysis in senescent T cells is supported by reduced pyruvate dehydrogenase kinase activity and the downregulation of Sirtuin1 [209]. In senescent Tregs, glucose consumption is accelerated, supporting their function. TLR8 signaling restored metabolic control and Treg function, improving antitumor immunity in vitro and in vivo [210]. Fewer studies have addressed senescence-associated changes in B-cell metabolism. Kurupati et al. reported that circulating switched memory B cells from aged people present high mitochondrial mass and ROS and lower FOXO1, a transcription factor controlling metabolic homeostasis in response to oxidative stress [211]. As a consequence, aged B cells exhibit a strong reduction in oxidative phosphorylation after activation. Moreover, it has been reported that DN B cells from the elderly utilize higher amounts of glucose and aerobic glycolysis to support survival and function [212]. Finally, reduced respiratory capacity due to insufficient NAD levels and decreased mitochondrial function (reduced ATP and increased ROS) have been described in monocytes and macrophages from elderly individuals [213].

Similarly, chronic infections can induce metabolic alterations in infected cells both indirectly, by altering helper cell function as described above, or directly, causing T-cell exhaustion. For instance, Kaposi’s sarcoma-associated herpesvirus (KHSV) has been shown to alter infected host cell glucose metabolism in infected cells by upregulating HIF1α, reducing mitochondrial capacity and releasing micro-RNAs, which are able to guide metabolic reprogramming, into the microenvironment [214]. HIV increased glycolysis and Glut1 expression in T cells. In Glut1^hi^ CD4^+^ T cells, viral transcription is linked to cholesterol metabolism via SREBP2 and to enhanced fatty acid synthesis [215]. Similarly, increased glucose metabolism and surface Glut1 and Glut 3 expression have been reported in monocytes from HIV subjects [216]. In infected B cells, EBV induced the activation of the PI3K/Akt pathway and enhanced HIF-1α expression, increasing glucose uptake and glycolytic flux [217]. HBV impacts the infected host cell’s metabolism by upregulating the expression of glucose transporters (GLUT) through mTOR signaling and promoting glycolysis [218]. Interestingly, it has been reported that Mtb also perturbs metabolic circuits and alters effector functions in lung CD8^+^ T cells. As Mtb infection become chronic, mitochondrial metabolism deteriorates in CD8^+^ T cells, resulting in an increased dependency on glycolysis that, in turn, potentiates inflammatory cytokine production [219]. Overall, the evidence that senescent immune cells rely on metabolic reprogramming for survival and activity suggests that their state could be reversed by manipulating cell metabolism, offering therapeutic strategies for the treatment of infections.

## 6. Targeting of Senescent Immune Cells

In line with the increased understanding of the immune system during aging, in the last years, different potential therapeutic approaches to counteract immune dysfunctions in the elderly have been proposed [6]. In this review, we focus on approaches targeting cellular senescence, whereas details and more extensive information on other immune-targeting approaches can be retrieved elsewhere [57].

Senotherapeutics are a new class of drugs that consists of two members, senolytics and senomorphics, with the goal of removing senescent cells and/or blocking the SASP [220]. Senolytics classically relied on the use of apoptosis-inducing drugs, yet the harnessing of innate and humoral immunity, targeting specific receptors, has been proposed more recently as an attractive strategy to stimulate senescent cell clearance. Senomorphics act as SASP inhibitors without cell killing and without compromising cell cycle arrest. These drugs primarily target pathways related to SASP expression and transcription factors. In this context, metabolic or epigenetic targeting has also been proposed as a means of reverting the state of cellular senescence. A second approach is based on the neutralization of the activity and function of specific SASP factors, such as cytokines, with specific antibodies [220].

Most of these approaches have shown efficacy in modulating the senescence of cells from multiple tissues, with a bystander benefit for the aged immune system and for the treatment of a variety of diseases of old age [221,222]; fewer studies have described their direct effectiveness in senescent immune cells. Here, we discuss this evidence, considering their application shown in the context of aging or immunity against infection (Table 1).

### 6.1. Therapeutic Opportunities for Aging

The main hurdle in eliminating senescent immune cells is the fact that exclusive criteria for discriminating immunosenescent cells from highly differentiated but non-senescent immune cells are still largely missing. Although not completely validated, immunological markers for senescent immune cells have been proposed and might provide additional therapeutic options in addition to traditional markers. The classical senescent marker, an increase in SA-β-gal activity, was observed in many aged subsets of peripheral blood mononuclear cells, including T and B lymphocytes, plasmacytoid dendritic cells (pDCs), natural killer (NK) cells and monocytes [38], whereas it might not be reliable enough to completely differentiate senescent from activated macrophages [223]. The use of a prodrug processed into a cytotoxic compound by β-gal has been demonstrated to be extremely effective in dampening inflammation and restoring physical function in aged mice, with this effect being partly mediated by the depletion of SA-β-gal-positive macrophages [224]. Thus, although this strategy could suffer from off-target effects, lysosomal β-gal can be effectively leveraged to selectively eliminate immunosenescent cells [225]. A study recently reported the development of a CD153 peptide vaccine able to induce the production of anti-CD153 antibodies and to decrease the number of senescent CD153^+^ T cells infiltrating the adipose tissue of obese mice [225]. Notably, vaccinated animals showed improved glucose tolerance and insulin resistance [226]. On the other hand, this approach may be contraindicated in the case of mycobacterial infection [227]. In addition, the clinical translation of vaccination strategies targeting senescence epitopes may require careful consideration, especially considering their lasting effects, which might be difficult to reverse. The p16^INK4a^ tumor suppressor inhibits the cell cycle, promotes cellular senescence and has been linked to aging in mammalian systems. In particular, p16^INK4a^-expressing T cells accumulate in human peripheral blood with aging, correlating with plasma interleukin-6 concentration, a marker of human frailty [228], and p16+ T cells seem to have a reduced immune capacity, as the specific deletion of p16 in these cells enhances immune function in aged mice [229].

Many of the strategies to modulate SASP target key activation pathways, such as mitogen-activated protein kinase (MAPK) signaling, phosphoinositide 3-kinase (PI3K) and mammalian target of rapamycin (mTOR) signaling, which lead to NF-kB activation [220]. The p110 Delta subunit of phosphoinositide 3-kinase (PI(3)K), an upstream activator of mTOR, is selectively expressed in leukocytes and is critical for lymphocyte biology. Patients with activated PI3K Delta syndrome present a dominant mutation in the PI3K catalytic subunit p110d, resulting in T-cell senescence and immunodeficiency [230]. Notably, the administration of a selective PI3Kd inhibitor, Leniolisib (CDZ173), reduced senescent T cells and decreased inflammatory markers in ongoing clinical trials [231,232]. The inhibition of PI3-kinase also corrected the aberrant migration seen in old neutrophils [73]. The switch to NK-like activity has been described in senescent CD8+ T cells during aging. The increased expression of the NK receptor and the concomitant decrease in TCR levels is mediated by sestrins, a family of stress-sensing proteins, and their blockade increased telomere activity, restored T cell function and enhanced the immune response to vaccination in aged mice [29,30]. Importantly, the silencing of sestrin expression in primary T-cell populations from old humans similarly enhanced antigen-specific proliferation and cytokine production in vitro via p38 MAPK inhibition [29]. Moreover, p38 MAPK blockade reversed CD8^+^ T-cell senescence via an mTOR-independent pathway, increasing autophagy [31]. Altered p38 activity is also associated with a reduction in TIM-4 receptor expression on aged senescent macrophages, leading to impaired efferocytosis [122]. Administering a p38 inhibitor rescued TIM-4 expression and restored a macrophage resolution phenotype both in vitro and in vivo in humans [122].

Senescent cells are “hypermetabolic”, and this metabolic reprogramming may potentially be therapeutically targetable [233], though it remains unclear how metabolism-targeted drugs can achieve sufficient specificity for senescent over non-senescent cells to allow clinical translation. Dietary interventions have the potential to limit the accumulation of senescent cells with age. Calorie restriction (CR) was one of the first interventions identified and able to extend lifespan [234]. CR impinges on several pathways, including mTOR inhibition, sirtuin activation and enhanced AMP kinase function, which in turn induces autophagy [235,236]. CR lowered markers of senescence in T cells, whereas the effect on NK cells remains controversial [237,238]. Several metabolic regulators and CR mimickers have been identified and tested for their anti-aging effects mediated by mTOR/AMPK pathway inhibition. The inhibition of this pathway could increase lifespan and health during aging in pre-clinical models and also reduce the incidence of respiratory tract infections in mice and augment the type I IFN response [239]. However, in aged people with increased senescent PD-1^+^ T cells, the mTORC1 inhibitors rapamycin and its analogs showed controversial results in enhancing immune function and reducing respiratory illness [240,241,242]. Further work is needed to evaluate rapamycin efficacy in reducing the clinical effect of aging and to investigate whether mTOR inhibition could be useful in preventing respiratory tract infections in specific subpopulations or whether inhibition is more effective against specific types of viruses. The results could help in the evaluation of drug repurposing, which has been proposed for patients affected by severe SARS-CoV-2 infection [243,244,245]. Another CR agent, Metformin, was able to enhance T-cell autophagy, normalize mitochondrial function and alleviate senescence-associated inflammation [246]. Metformin also extended the health span and lifespan in multiple animal models, both alone and in combination with rapamycin [247,248]. Resveratrol, a natural polyphenol compound, is drawing interest for its anti-aging activities mediated by the reduction in reactive oxygen species (ROS), the inhibition of cyclooxygenase (COX) and the activation of many anti-inflammatory pathways, including the one controlled by Sirtuin-1 (Sirt1) [249]. Resveratrol functions as an immunomodulator. It has been shown to restore T-cell function by targeting the immune-checkpoint signaling molecules PD-1/PD-L1 and to activate NK cells through AKT- and mTORC2-mediated c-Myb upregulation [249]. Remarkably, resveratrol also induced apoptosis and cellular senescence in primary and cancer cells [250]. Finally, spermidine, an endogenous metabolite, had a potent effect in reducing B-cell senescence and was able to restore the age-related decline in autophagy and responses to vaccination and infection of CD8^+^ T cells in old mice [251]. Considering how the pathways regulating metabolism are linked to immune cell function and senescence, they could hold a high therapeutic potential for treating clinical conditions associated with aging.

The hallmarks of aging include genomic instability, telomere attrition and epigenetic alterations [10]. In particular, epigenetic control is essential to the healthy development of effector cells of the immune system [252,253,254,255]. It is therefore not surprising that epigenetic pathways are dysregulated in aged immune cells and are responsible for their altered transcriptional profiles [256]. In the last several years, various epigenetic drugs have been developed to target epigenetic changes in different diseases and contexts [257], and investigating their effects on immunosenescence may provide novel ways to restore functional protective immunity during aging. In this regard, recent work showed that therapeutic efforts to reverse T-cell exhaustion during infection may require new approaches that increase the epigenetic plasticity of exhausted T cells [258,259].

Interestingly, metabolism and epigenetics are closely linked, together affecting the aging of the immune system. For example, CR modulates the sirtuin protein family, one of the first known epigenetic enzymes and a key regulator of aging [260]. Moreover, Metformin regulates the activation of AMPK, which directly governs the activities of several epigenetic enzymes, such as HATs, HDACs and DNMTs [261]. Studies using metabolism-targeted drugs specifically evaluating these chromatin-modifying pathways are still warranted.

### 6.2. Therapeutic Opportunities for Persistent Infections

Blocking early senescence in exhausted T cells in patients with chronic viral infection may enhance their efficacy [262]. In this regard, antibody blockade of inhibitory receptors has shown promise in re-activating T-cells. Importantly, these strategies enable the reversal of metabolic dysfunction by restoring TCR signaling, which stimulates glycolysis-enhancing pathways. In vitro studies showed that blocking PD-1 pathways restored effector functions in patients’ HIV-specific CD8^+^ T cells, improving pathogen control [167]. Moreover, the in vivo administration of anti-PD-1 antibody expanded and increased the functionality of virus-specific CD8^+^ T cells, significantly reduced plasma SIV RNA, prolonged survival and reduced markers of immune activation in SIV-infected macaques [157,263]. Anti-PD-L1 antibody treatment reduced HIV-1 replication and also increased CD4^+^ T and CD8^+^ T cells in HIV-infected humanized mice [264].

More recently, single-dose anti-PD-L1 therapy was evaluated in healthy HIV-1-infected persons on suppressive ART therapy. In this study, Gay et al. reported enhanced HIV-specific CD8+ T-cell responses in the blood of a subset of patients, but without any effects on HIV viral load. This result can likely be explained by the low dosage of anti-PD-L1 antibodies used [265]. PD-1 blockade was also shown to be effective in increasing IFN-γ effector functions and the clearance of the HBV virus in an in vivo model of HBV [266]. In chronic HCV infection, anti-PD-1 therapy led to only a moderate response [267]. The direct in vivo blockade of CTLA-4 during chronic viral infections such as LCMV, SIV and HIV failed to decrease the viral load or increase T-cell functionalities [268,269]. Overall, this evidence suggests that targeting multiple inhibitory receptors may be more efficacious. In HCV infections, the concurrent blockade of CTLA-4 and PD-1 reinvigorated HCV-specific CD8^+^ T cells in a CD4^+^ T-cell-independent manner [270]. A combination of anti-PD-1 and anti-CTLA4 therapies elicited better control of EBV infection, increasing EBV effector responses and decreasing EBV-infected B cells [271]. An additional promising target is P-Selectin Glycoprotein Ligand 1, PSGL-1. Its knockdown led to the downregulation of several inhibitory receptors, including PD-1, CD160, TIM-3, BTLA and LAG-3, improving antiviral responses [272]. LAG-3 expression has been associated with reduced LCMV-specific CD8 T-cell responses, and its blockade, in combination with anti-PD1/PD-L1 therapy, restored immune activity [273,274]. Moreover, LAG3 inhibition was shown to be effective in chronic HBV infection [275]. Unlike anti-PD-1 treatment, TIM-3 blockade has been reported to restore T cells from exhaustion and reduce Mtb replication within macrophages, thus improving outcomes in tuberculosis infection. Further, double blocking PD-1 and TIM-3 receptors increased the secretion of IL-1β and promoted Mtb clearance [276].

In addition to inhibitory receptors, cytokines have been identified as potential targets to reinvigorate exhausted T cells. IL-10 was positively associated with immune evasion and the persistence of viral infections such as HCV, HIV and EBV [277]. IL-10 blockade was effective in inhibiting viral persistence and enhancing T-cell functions during LCMC infection [278]. Consistently, IL-10Rα blockade resulted in the markedly increased secretion of IFN-γ by HIV-specific CD4^+^ T cells. However, combining IL-10 and PD-1 blockade could only partially restore T-cell-driven cytokine production [279]. On the contrary, in a mouse model of LCMV infection, combined anti-IL-10 and anti-PD-1 therapy synergistically enhanced the antiviral response of T cells [280]. Despite its benefits, there are several concerns regarding the use of anti-IL-10 therapy related to its role in maintaining oral tolerance and the potential emergence of undesirable side effects. Grint et al. demonstrated that IL-2 treatment successfully decreased the virus RNA levels in HCV in HCV/HIV-co-infected patients [281]. Targeting the type I IFN response in combination with ART was effective in reducing viral loads and restoring CD8^+^ T-cell functions in humanized mice with chronic HIV infection [282].

Several reports have suggested that metabolic deregulation contributes to T-cell exhaustion [158]. Bengsch et al. demonstrated that during chronic LCMV infection, PD-1 signals inhibited glycolytic and mitochondrial metabolism through peroxisome proliferator-activated receptor gamma coactivator 1-alpha (PGC1α) in early effector CD8^+^ T cells. Interestingly, the overexpression of PGC1α corrected some metabolic alterations in exhausted T cells and improved their effector function [283]. Altered mitochondrial metabolism was also described in exhausted HBV-specific T cells. Interleukin (IL)-12 recovered the HBV-specific T-cell effector function, increased their mitochondrial potential and reduced their dependence on glycolysis [284]. Finally, Metformin rejuvenates mitochondrial metabolism in T cells from Mtb-infected mice [219].

## 7. Conclusions and Future Perspectives

The immune system involves the interplay of many different cell types, collectively providing protection against infectious pathogens. Aging affects immune cell functions, resulting in increased severity of infections, enhanced predisposition to their complications and increased mortality in older people. The induction of senescence in immune cells can also occur in response to chronic viral infections, though information regarding a link between bacterial infections and immune senescence is emerging [197]. Despite the increased interest in developing therapeutic and preventive strategies targeting senescence in immunity, many of the underlying molecular processes, both intrinsic and extrinsic, are still not completely understood. In fact, whether the observed decline in immune function is a cause or a consequence of the cross-talk among various systems within organismal aging is still not well understood.

Mouse models have been particularly informative in aging studies; however, it remains to be established to what extent these models faithfully recapitulate the mechanisms underlying senescence processes in humans and their effects. Novel tools to track small populations of peripheral blood immune cells from clinical samples have enabled studies of cell exhaustion leading to senescence directly in humans, especially during chronic infections. Nonetheless, these studies did not consider potential changes in senescent cells residing in tissues. A combination of multiple scientific approaches (e.g., multiparametric analyses, high-resolution omics-technologies, system biology and big data analyses) would allow a better understanding of immune senescence during human aging and its outcomes for infections.

Targeting senescent cells has become an attractive alternative therapy for treating aging-related immune dysfunction, thus preventing or reducing the impact of different diseases in the elderly. Senotherapeutic strategies to eliminate senescent cells or disrupt their impact have achieved promising results in a variety of contexts. SASP modulators have been actively exploited to reduce mortality in hospitalized COVID-19 patients based on their potential to counteract the detrimental cytokine storm [285]. Future studies are needed to determine whether SASP modulators can also be beneficial for people suffering from chronic viral infections, such as HIV. However, some of them may have negligible direct effects on immune cells [286]. Senolytics typically target senescent cell anti-apoptotic pathways, survival pathways utilized by senescent cells to maintain their viability. Interestingly, ABT-263 has been shown to sensitize macrophages infected with a variety of virus types (influenza, herpes simplex and measles) to undergo cell death [287]. However, it is not known whether the drug was effective on infected senescent cells. The development of CAR-T cells has shown efficacy in cancer therapy [288] and autoimmune diseases [289,290]. In addition, recent evidence suggests their potential application as senolytic agents during aging for the treatment of age-associated diseases [291]. The use of CAR-T cells directed against senescence-specific surface antigens would open new possibilities to modify the immune system to restore active immune surveillance. Furthermore, CAR-T cells might be modified to be more resistant to their own exhaustion [292]. Strategies based on immune-mediated clearance should consider immune evasion mechanisms that senescent cells have developed to prevent their removal [293,294].

Studies in animal models in which senotherapeutics were tested have revealed various beneficial effects, but they also experienced adverse side effects [295]. Consistently, strategies proposed for SASP inhibition might result in off-target side effects affecting the immune system, such as neutrophilia, nephrotoxicity and changes in the T-cell phenotype, due to the close interconnection among the various pathways involved in inflammatory signaling [296]. Specifically, NF-kB pathway inhibition, either by genetic deletion or small molecules, has been shown to inhibit the development of mature lymphocytes and lymphoid progenitors [296,297]; to induce apoptosis of thymocytes, macrophages and B cells [298,299]; to impair lymphocyte growth and cytokine production [300]; and to potentially increase susceptibility to various infections [301]. Interestingly, the combination of Dasatinib, a kinase inhibitor, and quercetin, a flavonoid targeting the PI3K/AKT pathways, has been proven effective in pilot clinical trials in reducing senescent cells and alleviating physical dysfunction in patients with pulmonary fibrosis [302,303]. Despite this important proof of concept demonstrating the feasibility of clinical translation, larger controlled trials are needed to fully assess the efficacy of the therapy by identifying biomarkers of senescence, the potential off-target effects of the drugs and their mechanisms of action in humans before moving to age-related diseases.

The identification of specific intracellular and extracellular markers of cellular senescence will be instrumental in developing next-generation senotherapeutics characterized by increased selectivity and reduced off-target effects. In this regard, recent studies corroborated the use of nanoparticles for the specific delivery of drugs into senescent cells in vivo [225]. This approach may help to overcome the problem of specificity in conditions requiring chronic administration.

Given the complexity of the immune system and aging, targeting multiple components of the immune system at once could be required. This would need an extremely balanced approach to avoid undesired side effects. Indeed, while the strong stimulation of the immune system to recover its complete functionality could lead to acute inflammation or autoimmune diseases, exaggerating immune suppression to reduce inflammaging could result in increased susceptibility to infections. Future challenges will be, therefore, to identify the right combination of the various therapeutic approaches and to select the group of patients that would benefit the most. Finally, the large differences between individuals, particularly in the heterogeneity of immune system changes with age, must be considered. Thanks to multiomics analyses that provide personalized genetic and epigenomic information, precision medicine is now the gold standard approach in oncology, where treatments are decided based on the mutational profile of the patient [304]. In aging, distinct individuals respond differently to Metformin, with some developing side effects [305]. This evidence suggests that specific genetic or epigenetic characteristics might influence treatment efficacy. Stratification of the aged population based on biomarkers identified from multiomics data could help in recognizing subgroups of patients who would benefit most from specific treatments, thus allowing more tailored approaches.

## Figures and Tables

**Table 1:** Main potential therapeutic strategies to target senescent immune cells with implication for ageing and immunity against infections.

Approach	Target (molecular/cellular)	Experimental outcome	Benefits	Limitations	Ref.
***Senolytics***					
**CD153 peptide** (vaccination)	CD4+ CD44high CD62Llow PD-1+ CD153+ cells in VAT tissue	Elimination of infiltrating senescent T cells in adipose tissue in obese mice	Improved glucose tolerance and insulin resistance	Contraindicated in case of mycobacterial infections	[226,227]
**Prodrug SSK1** (oral administration)	SA- β -gal positive cells	Clearance of senescent macrophages	Dampened inflammation and restored physical function in aged mice	Off-target effects	[224]
**Genetic deletion**	p16(INK4a) in lymphocytes	Improved T cell immune function	Ameliorated several aging phenotypes	B lineage-specific ablation was associated with a markedly increased incidence of systemic, high-grade B-cell neoplasms	[229]
***Senomorphics***					
**Leniolisib** (oral administration)	PI3Kd	Reduction in PD-1+CD4+ and senescent CD57+CD4− T cells	Improved immune dysregulation and decreased fatigue in ongoing clinical trials	Potential genomic instability by augmenting off-target activity of activation-induced cytidine deaminase	[231,232]
**Genetic inhibition**	Sestrins in T cells	Restored T cell proliferation and cytokine production via p38 MAPK inhibition in senescent-like CD27−CD28−CD8+ T cells.	Enhanced vaccine responsiveness in old mice; enhanced immune function in primary human T cells	Prolonged inhibition of sestrins may result in malignancy	[29,30]
**Losmapimod** (oral administration)	p38 MAPK	Reduced systemic symptoms of inflamm-ageingr; escued TIM-4 expression; cleared apoptotic bodies and restored efferocytosis in macrophages	Improved skin inflammation resolution in aged people	Tested on a small cohort of individuals	[122]
**BEZ235** (oral administration)	PI3K/mTOR	Augment the type I IFN response; reversed infection-induced changes in metabolism	Increased lifespan and health and reduced the incidence of respiratory tract infections in mice	Controversial results obtained in humans	[239,240,241,242]
**Metformin** (oral administration)	AMPK	Enhanced T cell autophagy, normalized mitochondrial function, and alleviated senes-cence-associated inflammation	Extended health span and lifespan in multiple animal models	Focus on CD4 T cells as sources of inflammageing; studies in humans not conclusive	[246,247,248]
**Resveratrol** (oral administration)	SIRT1	Reduced ROS, inhibited COX, and activated anti-inflammatory pathways (Sirt1)	Anti-ageing in human trials; Restored T-cell function and NK cell activities	Possible risks related to nephrotoxicity	[249]
**Spermidine** (oral administration)	eIF5A	Reduced B cells senescence; improved autophagy in T cells	Restored response to vaccination and infection of CD8+ T cells in old mice	Toxic at high dose	[251]
***Antibodies against cytokines***					
**Anti-IL10**	IL-10	Enhanced T-cell functions	Inhibited viral persistence during LCMC infection in mice	Potential side-effects due to its role in immune tolerance	[278]
**Anti-IFN-I**	IFN receptor 2	Decreased T cell exhaustion marker expression, restored viral-specific CD8 T cell function	Decreased viral replication in conjunction with ART treatment in HIV-infected humanized mice	Potential side-effect due to different role of IFN-I signaling during acute and chronic infection	[282]
***Inhibitory receptors blockade***					
**Anti-PD-1**	PD-1	Reduced exhaustion, expanded and increased functionality of virus-specific CD8+ T cells,	Significantly reduced plasma SIV RNA and prolonged survival in SIV-infected macaques	Potential side-effect related to break of tolerance	[157,263]
		Reversed the exhausted phenotype, Increased IFN-g effector functions	Clearance of HBV virus in an in vivo model of HBV		[266]
**BMS-936558**		No substantial changes in immune phenotype	Reduced viral load in a subset of HCV patients enrolled in the trial	Immune-related adverse events of mild-to-moderate intensity	[267]
**Anti-PD-L1**	PD-L1	Restored viral-specific T cell functions	Reduced HIV-1 replication in HIV-infected humanized mice	No effect on HIV viral load in humans, likely due to low dosage used.	[265]
**Anti-CTLA-4**	CTLA-4	Enhanced viral specific responses	Better control of EBV and HIV infections in combination with anti-PD-1	No effect in viral infection when used as monotherapy	[270,271]

## Data Availability

Not applicable.

## Abbreviations

AKT	Protein kinase B
ATM	Ataxia Telangiectasia Mutated
BCL-xl	Bcl-2-Like Protein-1
BCR	B-cell receptor
B-gal	B-galactosidase
BTLA	B- and T-Lymphocyte Attenuator
CAR-T	Chimeric Antigen Receptor-T
CD122	Interleukin-2 receptor-b
CD127	Interleukin-7 receptor
CDKN	Cyclin-Dependent Kinase inhibitor
CMV	Human cytomegalovirus
CR	Calorie restriction
CTLA-4	Cytotoxic T-lymphocyte antigen-4
CXCR	C-X-C Motif Chemokine Receptor
DDR	DNA damage response
DNMTs	DNA methyltransferases
EBV	Epstein–Barr virus
ERK	Extracellular signal-regulated kinase
FOXP3	Forkhead box P3
gH2AX	Gamma H2A histone family member X
HAT	Histone Acetyltransferases
HBV	Hepatitis B virus
HCV	Hepatitis C virus
HDAC	Histone Deacetylases
HIV	Human immunodeficiency virus
HHV-8	Human herpesvirus-8
HLA-DR	Human Leukocyte Antigen DR
HSV-1/2	Herpes simplex-1/2 virus
HTLV-I	Human T-cell leukemia virus type-1 (HTLV-I)
JNK/AP-1	c-Jun N-terminal kinase/activator protein-1
KLRG1	Killer Cell Lectin-Like Receptor G1
LAG-3	Lymphocyte activation gene 3
LC3	Microtubule-associated *protein* 1A/1B-light chain 3
LCMV	Lymphocytic choriomeningitis virus
LPS	Lipopolysaccharide
MAP	Mitogen-activated protein
Mtb	Mycobacterium tuberculosis
mTOR	Mammalian target of rapamycin
NKCC	*Natural Killer* Cellular Cytotoxicity
NET	Neutrophil extracellular trap
NLRP3	NLR family pyrin domain containing 3
PD-1	Programmed cell death protein 1
PI3K	Phosphatidylinositol 3-kinase
PTEN	Phosphatase and Tensin Homolog
ROS	Reactive oxygen species
RSV	Respiratory syncytial virus
SARS-CoV-2	Severe acute respiratory syndrome coronavirus 2
SASP	Senescence-Associated Secretory Phenotype
SIV	Simian immunodeficiency virus
STAT	Signal Transducer and Activator of Transcription
TCF1	T Cell Factor 1
TCR	T-cell receptor
TFH	T follicular helper
TIGIT	T-cell immunoglobulin and ITIM domain
TH	T helper
TIM-3	T-cell immunoglobulin and mucin-domain containing-3
TLR	Toll-Like Receptor
TNF	Tumor Necrosis Factor
VZV	Varicella-zoster virus
